# Post-traumatic stress disorder and post-traumatic stress symptoms following critical illness in medical intensive care unit patients: assessing the magnitude of the problem

**DOI:** 10.1186/cc5707

**Published:** 2007-02-22

**Authors:** James C Jackson, Robert P Hart, Sharon M Gordon, Ramona O Hopkins, Timothy D Girard, E Wesley Ely

**Affiliations:** 1 Clinical Research Center of Excellence (CRCOE), VA Tennessee Valley Geriatric Research, Education and Clinical Center (GRECC), 1310 24. ^th ^Avenue, S., Nashville, TN 37212, USA; 2 Division of Allergy/Pulmonary/Critical Care Medicine, Vanderbilt University, T1218 Medical Center North, Nashville, TN 37232-2650, USA; 3 Center for Health Services Research, Vanderbilt University, 6100 Medical Center East, Nashville, TN 37232-8300, USA; 4 Department of Psychiatry, 1601 23^rd ^Avenue, South, Vanderbilt University School of Medicine, Nashville, TN 37212, USA; 5 Department of Psychiatry, West Hospital, 1200 E. Broad, VCU Medical Center, Richmond, VA 23298, USA; 6 VA Tennessee Valley Geriatric Research, Education and Clinical Center (GRECC), 1310 24^th ^Avenue, S., Nashville, TN 37212, USA; 7 Psychology Department and Neuroscience Center, 1082 SWKT, Brigham Young University, Provo, UT 84602, USA; 8 Department of Medicine, Pulmonary and Critical Care Division, LDS Hospital, Eighth Avenue and C Street, Salt Lake City, UT 84113, USA

## Abstract

**Introduction:**

Post-traumatic stress disorder (PTSD) is a potentially serious psychiatric disorder that has traditionally been associated with traumatic stressors such as participation in combat, violent assault, and survival of natural disasters. Recently, investigators have reported that the experience of critical illness can also lead to PTSD, although details of the association between critical illness and PTSD remain unclear.

**Methods:**

We conducted keyword searches of MEDLINE and Psych Info and investigations of secondary references for all articles pertaining to PTSD in medical intensive care unit (ICU) survivors.

**Results:**

From 78 screened papers, 16 studies (representing 15 cohorts) and approximately 920 medical ICU patients met inclusion criteria. A total of 10 investigations used brief PTSD screening tools exclusively as opposed to more comprehensive diagnostic methods. Reported PTSD prevalence rates varied from 5% to 63%, with the three highest prevalence estimates occurring in studies with fewer than 30 patients. Loss to follow-up rates ranged from 10% to 70%, with average loss to follow-up rates exceeding 30%.

**Conclusion:**

Exact PTSD prevalence rates cannot be determined due to methodological limitations such as selection bias, loss to follow-up, and the wide use of screening (as opposed to diagnostic) instruments. In general, the high prevalence rates reported in the literature are likely to be overestimates due to the limitations of the investigations conducted to date. Although PTSD may be a serious problem in some survivors of critical illness, data on the whole population are inconclusive. Because the magnitude of the problem posed by PTSD in survivors of critical illness is unknown, there remains a pressing need for larger and more methodologically rigorous investigations of PTSD in ICU survivors.

## Introduction

Estimates of post-traumatic stress disorder (PTSD) prevalence in critically ill cohorts are reported to be as high as 63% [1] and exceed or rival those of traditionally 'high-risk' populations as well as populations with medical disorders such as cancer and myocardial infarction [2,3] (Table 1). It may be that critical illness is uniquely stressful due to factors associated with the intensive care unit (ICU) experience such as awareness during painful procedures, a sense of helplessness, loss of control, and an imminent threat of death. Such experiences may be 'traumatic' as trauma is a generic term that can refer to experiences that are physical and/or psychological in nature. Alternatively, it may be that the limited research conducted to date has substantially overestimated the prevalence of PTSD after critical illness or that PTSD in ICU survivors is qualitatively different than that resulting from war, natural disasters, or other types of traumatic stressors. A comprehensive evaluation of this and other issues is timely and important as concern about PTSD among ICU survivors is growing and has led, in some cases, to changes in the delivery of care and in the management of patients in response to the perception that PTSD is a common outcome.

**Table 1:** A comparison of PTSD prevalence rates across 'at-risk' adult populations

Traumatic event^a^	No. of studies	Range of prevalence estimates	Comments
Rape [56,57]	>50	14%–80%	Completed rape is associated with the greatest risk of PTSD.
Man-made disaster [58]	106	25%–75%	Studies with highest prevalence estimates were conducted on subjects exposed to 'extreme' trauma shortly after the event.
ICU	16	5%–63%	Prevalence rates are extremely high relative to other medical populations.
Natural disaster [58]	86	5%–60%	Most studies report rates in the lower half of the 5%–60% range.
Political refugee experience [59]	22	4%–44%	Prevalence rates may be affected by the use of tools possibly insensitive to cultural expressions of PTSD.
Cancer survivors [60]	>100	1.9%–39%	Prevalence rates are quite controversial due to debate over status of cancer as a traumatic stressor.
MVA survivors [61]	>100	7.6%–34%	Many MVA survivors have histories of prior trauma, thus PTSD symptoms may be pre-existing.
MI survivors [62]	4	0%–16%	Prevalence studies are limited and have small sample sizes.
Combat in Vietnam [63,64]	>100	1.8%–15%	Prevalence estimates of subpopulations of Vietnam veterans (such as those injured in combat) are higher than 15%.

^a^Studies listed are either recent reviews or key investigations of the topic which include a discussion of prevalence. ICU, intensive care unit; MI, myocardial infarction; MVA, motor vehicle accident; PTSD, post-traumatic stress disorder.

A number of recent reviews have looked at the association between medical illness and the development of psychiatric illness [4-6]. However, no review has focused exclusively and/or comprehensively on PTSD following medically related critical illness. With this review, we sought to accomplish four goals: (a) to evaluate existing research pertaining to PTSD following medically related critical illness, with a primary focus on prevalence, (b) to provide a critical analysis of methodological characteristics of the studies under review, (c) to provide a summary of possible explanations for PTSD following critical illness, and (d) based upon an analysis of the strengths and weaknesses of existing investigations, to offer recommendations for future research. For a definition of PTSD, see Table 2.

**Table 2:** DSM-IV definition of post-traumatic stress disorder

Definition of post-traumatic stress disorder^a^
A potentially debilitating psychiatric condition that develops as the result of being exposed to a traumatic occurrence 'in which a person experienced, witnessed, or was confronted with an event or events that involved actual or threatened death or serious injury, or a threat to the physical integrity of self or others' and which generates 'intense feelings of fear, helplessness, or horror' in those exposed to the trauma. This condition is characterized by a constellation of symptoms in three domains:
A. Symptoms of re-experiencing (for example, intrusive thoughts and upsetting recollections of the trauma, recurrent dreams or nightmares, and flashbacks).
B. Symptoms of avoidance and emotional numbing (for example, efforts to avoid conversations, places, and thoughts associated with the trauma; detachment from others; and a restricted range of affect).
C. Symptoms of increase arousal (for example, sleep disruption, hypervigilance, and exaggerated startle response).
These symptoms must meet two criteria to satisfy diagnostic criteria:
1. Symptoms must cause significant impairment in social, occupational, or other important functional domains.
2. Symptoms must be present for at least 1 month after exposure to the traumatic event or events.

^a^Definition obtained from the *Diagnostic and Statistical Manual of Mental Disorders, Fourth Edition *(DSM-IV).

## Materials and methods

### Study identification and selection

A literature search for all articles pertaining to critical illness and PTSD was conducted using both the Psych Info and US National Library of Medicine MEDLINE databases. Key words/phrases used to search these databases included 'post-traumatic stress disorder' AND 'critical illness' (25 abstracts via MEDLINE and 5 via Psych Info) or 'post-traumatic stress disorder' AND 'intensive care' (81 abstracts via MEDLINE and 19 via Psych Info). Reference lists from identified articles were used to identify any additional studies.

### Study inclusion criteria and evaluation

For inclusion in this review, studies were required (a) to evaluate the association between medical ICU hospitalization and PTSD (either the diagnostic entity called PTSD or post-traumatic stress symptoms [PTSS]) and (b) to employ qualitative and/or objective measures of PTSD or PTSS. Investigations published in a language other than English were excluded as were unpublished studies and abstracts. One of the authors (JCJ) reviewed all of the articles in question to ensure that they met the above criteria.

### Data extraction and analysis

The following aspects of each study were identified, abstracted, and analyzed: study population, study design, timing of evaluations, study aims, exclusion criteria, methods of assessing PTSD, and all relevant results compared across study populations, including follow-up rates. All individual articles were assigned a 'quality rating' according to the Oxford Centre for Evidence-Based Medicine guidelines for symptom prevalence studies [7]. Ratings ranged from 1 to 3, with lower numbers indicating higher quality.

## Results

### Search for articles

A total of 78 non-overlapping potential abstracts were identified in the search of the databases and reference lists (the most recent search was performed in October 2006). Of these, 16 papers met inclusion criteria (Table 3). A number of studies consisting *entirely *of physical trauma and/or surgical ICU patients were identified and excluded from review due to the likelihood that the PTSD symptoms experienced by these patient populations could have been generated by either trauma-related injuries or surgical interventions. The authors recognize that trauma and surgical ICU patients may be similar in many respects to their medical ICU counterparts and, indeed, they may have overlapping experiences. Nevertheless, we chose to exclude such patients so as to focus as specifically as possible on the unique contributions of *medically related *critical illness to the development of PTSD. Similarly, a number of research investigations of medical ICU survivors assessing anxiety or memories of the ICU generically were identified and were also excluded as they did not include a specific focus on PTSD or PTSS. One investigation evaluated PTSD symptoms after critical illness but did not include data regarding prevalence rates and thus was excluded [8].

**Table 3:** Studies that report the prevalence of PTSD in medical ICU patients

Study	Population	Design	Quality rating^a^	Number lost to follow-up^b^	Follow-up time point	Tool	Rate of PTSD or PTSS	Risk factors
Rattray *et al*., 2005 [16]	General medical ICU	Prospective cohort	2b	109 enrolled at discharge, 87 at 6 months, 80 at 12 months; 27% lost to follow-up	Hospital discharge, 6 months, and 12 months	IES	20% with high avoidance scores and 18% with high intrusion scores	Avoidance and intrusive symptoms related to younger age, 'frightening' ICU experience, APACHE II scores, ICU/hospital lengths of stay, and recall of experiences
Capuzzo *et al*., 2005 [9]	General medical ICU	Prospective cohort	2b	84 at 1 week, 63 at 3 months; 25% lost to follow-up	1 week and 3 months	IES	5% with PTSS	PTSD symptoms associated with fewer factual memories
Cuthbertson *et al*., 2004 [10]	General medical ICU	Prospective cohort	2b	111 enrolled, 78 completed; 30% lost to follow-up	3 months	DTS	14% with PTSD	PTSD associated with younger age, length of mechanical ventilation, and previous psychiatric history
Nickel *et al*., 2004 [11]	General medical ICU	Cross-sectional	3b	41; percentage lost to follow-up not recorded	Unknown	PTSS-10, SCID	17% with PTSS; 9.76% with PTSD	PTSD associated with previous psychiatric history
Jones *et al*., 2003 [12]	General medical ICU	Randomized controlled trial	1b	126 eligible patients, 114 at 8 weeks, 102 at 6 months; 20% lost to follow-up	8 weeks and 6 months	IES	51% with probable PTSD at 6-month follow-up	Presence of delusional memories increased risk of PTSD symptoms
Kress *et al*., 2003 [13]	General medical ICU	Prospective cohort	2b	105 patients enrolled, 32 at follow-up; 70% lost to follow-up	~1 year	IES-R, clinical interview	18.5% with PTSD; 54% from control group; 0 from intervention group	Presence of delusional memories increased the risk of PTSD; sedative interruption decreased the risk of PTSD
Schelling *et al*., 2001^c ^[1]	General medical ICU	Retrospective cohort	2b	24 eligible, 20 completed testing; 16% lost to follow-up	21 to 49 months	PTSS-10, SCID	40% with PTSD (63% placebo group; 11% treatment group)	Administration of hydrocortisone related to a lower incidence of PTSD in ICU survivors
Scragg *et al*., 2001 [14]	General medical ICU	Cross-sectional	3b	142 eligible, 80 usable surveys returned; 44% lost to follow-up	>5 years	IES, TSC-33, ETIC-7	30% with PTSS; 15% with PTSD	Female gender/younger age associated with increased PTSD risk
Eddleston *et al*., 2000 [15]	General medical ICU	Prospective cohort	2b	227 available, 143 completed; 37% lost to follow-up	3 months	Selected PTSD questions	36% with 'distressing flashbacks'	Female gender related to increased risk of distressing flashbacks
Deja *et al*., 2006 [23]	ARDS survivors	Retrospective cohort	2b	129 enrolled, 65 at follow-up; 50.4% lost to follow-up	57 ± 32 months	PTSS-10	29% with 'high risk' of PTSD	PTSD associated with anxiety in the ICU; perceived social support related to decreased risk of PTSD
Kapfhammer *et al*., 2004 [17]	ARDS survivors	Retrospective cohort	3b	80 in the original study, 46 at follow-up; 42% lost to follow up	Median of 8 years	PTSS-10, SCID	43% with PTSD at discharge; 23.9% with PTSD at follow-up	PTSD was associated with greater ICU length of stay
Shaw *et al*., 2001 [20]	ARDS survivors	Cross-sectional	3b	20; N/A	Unknown	IES	35% with PTSS	Unknown
Stoll *et al*., 1999^d ^[18]	ARDS survivors	Retrospective cohort	3b	52; 35% lost to follow-up	Two time points at least 2 years apart (1 to 13 years after discharge)	PTSS-10, clinical interview	25% with PTSD	Greater number of traumatic memories associated with increased frequency and intensity of PTSD
Schelling *et al*., 1998^d ^[19]	ARDS survivors	Retrospective cohort	2b	80; 22% lost to follow-up	6 to 10 years, median 4 years	PTSS-10	27.5% with PTSD	Number of adverse experiences associated with higher PTSS-10 scores
Schelling *et al*., 1999^c ^[22]	Septic shock survivors	Retrospective cohort	2b	54; percentage lost to follow-up not recorded	2 to 9 years	PTSS-10, clinical interview	38% with PTSD (18.5% with PTSD in treatment group; 59% in control group)	PTSD associated with longer ICU treatment and increased number of traumatic experiences
Nelson *et al*., 2000 [21]	Acute lung injury survivors	Cross-sectional	3b	34 eligible, 24 completed; 29% lost to follow-up	6 to 41 months, mean 19 months	Seven items pertaining to PTSD	39% with 'bad memories or dreams'	Deeper levels of sedation and neuromuscular blockade exposure associated with increased risk of PTSD

^a^Quality of study methods was rated according to Oxford Centre for Evidence-Based Medicine guidelines and ranged from 1 to 3, with lower numbers indicating higher quality. Letters used to designate level 1 to 3 studies indicated gradations of quality ranging from 'a' (higher quality) to 'b' (lower quality). ^b^Total number of patients who were actual study participants as opposed to those who were simply enrolled; percentage lost to follow-up refers to the percentage of patients who for any reason did not participate in the follow-up portion or portions of the study. A few studies did not include follow-up components, thus loss to follow-up rates are not applicable (N/A). ^c^Fourteen patients in the 2001 study of Schelling *et al*. [1] had previously been in the 1999 investigation of Schelling *et al*. [22]. ^d^These investigations were conducted on the same population, and the follow-up evaluations in the 1999 study of Stoll *et al*. [18] occurred approximately 2 years after patients completed their participation in the 1998 study of Schelling *et al*. [19]. APACHE II, Acute Physiology and Chronic Health Evaluation II; ARDS, acute respiratory distress syndrome; DTS, Davidson Trauma Scale; ETIC-7, Experience of Treatment in the Intensive Care Unit-7; ICU, intensive care unit; IES, Impact of Events Scale; IES-R = Impact of Events Scale-Revised; PTSD, post-traumatic stress disorder; PTSS, post-traumatic stress symptoms; PTSS-10, Post Traumatic Stress Scale-10 for the Intensive Care Unit; SCID, Structured Clinical Interview for the *Diagnostic and Statistical Manual of Mental Disorders, Fourth Edition*; TSC-33, Trauma Symptom Checklist-33.

### Methods of reviewed articles

#### Subject characteristics

All investigations were conducted exclusively on adult critically ill patients. Studies focused on general medical ICU populations [9-16] as well as on critically ill patients with specific medical conditions such as ARDS/acute lung injury and septic shock [1,17-22]. Within individual studies, patients had significant variability with regard to key characteristics such as ICU length of stay, ventilation status and duration of mechanical ventilation, severity of illness, and the time to PTSD assessment. One investigation included patients with ICU lengths of stay from 11 to 99 days [22]. Another study included both patients with and without mechanical ventilation as well as those with APACHE II (Acute Physiology and Chronic Health Evaluation II) scores ranging from 4 to 38, suggesting extreme differences in illness severity [10]. In a third investigation, follow-up evaluations were conducted at intervals ranging from 1 to 13 years [18].

#### Study design

A total of six studies were prospective in nature; five of these were cohort studies [9,10,13,15,16] and one was a randomized controlled trial [12]. Six investigations employed a retrospective cohort design [1,17-19,22,23]. Four studies were cross-sectional [11,14,20,21]. Sample sizes were universally small, and the number of patients participating in follow-up ranged from 20 [1,20] to 143 [15] patients. Four studies evaluated individuals at multiple time points, and initial evaluations occurred within two months of hospital discharge and follow-up evaluations occurred at widely varying intervals of up to eight years [9,12,16,18]. The remaining investigations evaluated patients at a single time point, ranging from 3 months to 13 years after hospital or ICU discharge [1,10,11,13-15,17,19-23]. The percentage of patients lost to follow-up (for any reason) varied from 16% [1] to 70% [13], and the average rate of loss to follow-up was 32.5%. Three samples consisted of patients who were five or more years apart with regard to time from ICU or hospital discharge [18,19,22].

#### Exclusion criteria/identification of pre-existing psychiatric illness

Studies in which exclusion criteria were stated explicitly included prior psychiatric illness or neurologic trauma or disease [1,12,14,18,22]. Methods of identifying pre-existing psychiatric disorders varied widely across studies, and only five studies formally inquired about patients' pre-morbid psychiatric histories [10,11,13,17,22]. One of these investigations included a single question about pre-morbid psychiatric history, and this regarded whether subjects had seen a mental health professional or general practitioner for psychiatric reasons prior to ICU hospitalization [10].

#### Methods of assessing PTSD

A total of nine investigations relied solely on standardized brief screening tools in their assessment of PTSD or PTSS, including the Post-Traumatic Stress Scale-10 for the ICU (PTSS-10), Impact of Events Scale (IES), IES Revised, Davidson Trauma Scale (DTS), Trauma Symptom Checklist-33, and the Experiences of Treatment in the Intensive Care-7 [1,9,10,12,14,16,19,20,23]. With the exception of two investigations, these tests were administered in person [14,23]. Diagnoses of PTSD were repeatedly made entirely on the basis of information derived from screening tools. For example, Cuthbertson and colleagues [10] reported that 14% of their subjects met full diagnostic criteria for PTSD, despite the fact that the DTS (used in their investigation) is not a diagnostic tool. Similarly, Schelling and colleagues [19] diagnosed nearly 30% of ARDS survivors with PTSD on the basis of a cutoff score as opposed to a formal clinical interview. Few studies attempted to identify or quantify the clinical significance of PTSD or to evaluate commonly studied outcomes in this regard (for example, increased health care use, increased marital or family conflict, substance abuse, and days away from work), although three investigations did focus on the association between PTSD and health-related quality of life [17,19,22].

A total of five investigations relied on structured clinical interviews such as the Structured Clinical Interview for the DSM-IV (*Diagnostic and Statistical Manual of Mental Disorders, Fourth Edition*) (SCID) [11,13,17,18,22], employing them after screening tools were suggestive of probable PTSD. Generally, the use of more comprehensive tools such as the SCID resulted in the identification of fewer cases. For example, in the study by Nickel and colleagues [11], approximately half of the subjects identified as having PTSD via the PTSS-10 were false-positive according to the SCID.

### Primary findings

#### How prevalent is ICU-related PTSD?

Prevalence rates ranged from 5% to 63% and showed little variance regardless of whether the outcome in question was PTSD or PTSS; the three highest rates (54%, 59%, and 63%) occurred in investigations that purported to diagnose PTSD [1,13,22]. Importantly, these rates were reported in subpopulations (control groups) with sample sizes of between 11 and 27 patients and were higher than the rates reported in their entire populations. Prevalence rates varied depending on the time of assessment and were highest at the time of hospital discharge or shortly thereafter, decreasing over time. For example, Kapfhammer and colleagues [17] reported that 43.5% of study subjects had PTSD at hospital discharge whereas 23.9% suffered from PTSD an average of eight years later.

General medical ICU cohorts had both the lowest and highest rates of PTSD or PTSS compared with more specialized populations. In studies of general medical ICU patients, prevalence rates ranged from 5% [9] to 63% [1], and rates in specialized populations ranging from 18.5% [22] to 43% [17]. In the three studies comparing patients from different treatment conditions [1,13,22], marked differences in prevalence rates existed between 'treatment' and 'control' arms.

#### Risk factors for PTSD

Risk factors were not studied systematically across studies, although a number of risk factors were identified (Table 4). Two investigations reported that delusional memories (as opposed to factual ones) increased the risk of PTSD [11,12], and another study supported a relationship between fewer factual memories and a greater likelihood of PTSD [9]. Alternatively, three studies implicated factual memories in the development of PTSD [1,18,19], reporting an association between the number of traumatic memories and higher scores on PTSD screening tools. One study reported that greater recall of ICU-related experiences was associated with more intrusive symptoms [16]. One study reported an association between the presence of anxiety in the ICU and symptoms of PTSD [23]. Hospital- or treatment-related variables associated with PTSD or PTSS were associated with increased length of stay and/or duration of mechanical ventilation [10,16,17] as well as greater levels of sedation and/or neuromuscular blockade [13,21]. Hydrocortisone treatment was associated with a decreased risk of PTSD in two investigations [1,22].

**Table 4:** PTSD risk factors reported in the ICU- and PTSD-related literature at large

Known risk factors for PTSD or PTSD symptoms in the ICU
ICU length of stay (longer duration)
Hospital stay (longer duration)
Length of mechanical ventilation
Greater levels of sedation
Female gender^a^
Younger age^a^
Pre-existing psychiatric history^a^
Greater number of traumatic memories/frightening recollections^a^
Presence of delusional memories^a^

^a^Indicates established risk factors that have been identified in the general PTSD literature. ICU, intensive care unit; PTSD, post-traumatic stress disorder.

Demographic and historical variables associated with an increased risk of PTSD or PTSS included younger age [10,14,16], a prior mental health history [10,11], and female gender [14,15]. A greater degree of perceived social support was reported to be protective against the development of PTSS [23].

## Discussion

### Challenges to studying PTSD

As others have observed, PTSD, as concurrently conceptualized by the DSM-IV and the psychiatric community, is a complex condition that presents unique diagnostic challenges for clinical researchers [24]. Unlike virtually all other psychiatric conditions, which can be diagnosed solely on the basis of whether symptoms are present or absent, a diagnosis of PTSD requires exposure to a traumatic event or events. It often exists concurrently with other psychiatric disorders [25], making the relative contributions of each respective disorder to functional impairment potentially hard to discern. In medically ill populations, symptoms of PTSD are frequently expressed in nuanced and highly idiosyncratic ways and may not be captured through simple self-report questionnaires [4,26,27]. Additionally, self-report measures typically do not allow researchers to determine whether a constellation of symptoms reflect PTSD or a time-limited adjustment disorder [4]. For these and other reasons, the accurate identification of PTSD or PTSS in time-limited research contexts is a significant challenge. Although many investigations of PTSD following critical illness have used methodological rigor, the existing body of work on the subject has a number of significant limitations, as is often the case with early explorations in most arenas. These limitations raise questions about the prevalence rates of PTSD and the magnitude of the problem that PTSD represents to ICU survivors.

### Limitations of existing studies

As previously described, the methodological limitations of the aforementioned studies are significant and may have contributed to overestimates of PTSD or PTSS prevalence. In particular, the practice of using screening tools for diagnostic purposes is problematic. Certainly, screening tools and questionnaires vary widely in quality and comprehensiveness, and some self-report questionnaires possess fairly robust psychometric properties [28]. Nevertheless, such instruments are not typically intended to definitively identify the presence, absence, or severity of PTSD and tend to yield significantly higher false-positive rates than comprehensive diagnostic measures such as the SCID-PTSD and the Clinician-Administered PTSD Scale [29], although this is not always the case. A study of burn survivors conducted by Tedstone and Tarrier [30] may be instructive in this regard as it showed that whereas nearly 40% of their cohort were classified as 'PTSD cases' via the IES, only 2% were found to actually have PTSD when assessed with a comprehensive instrument, the Penn Inventory. Additionally, most screening tools have not been validated on patients with critical or life-threatening illness, thus responses to various questions may be confounded (for example, anticipating a 'foreshortened future' may be related to the experience of suffering from a particular medical condition and not a symptom of anxiety) [4,31]. Additionally, few screening tools assess DSM-IV criteria A (exposure to a traumatic stressor) and F (the presence of clinically significant impairment), although the positive endorsement of both criteria must occur for PTSD to be diagnosed. The failure to assess criteria A and F is problematic, particularly because the symptoms of PTSD reported by individual ICU survivors (and attributed to an episode of critical illness by researchers) could potentially be the result of exposure to prior traumatic stressors.

Although some may argue that critical illness and associated factors such as prolonged hospitalization and mechanical ventilation are always traumatic stressors, this is not necessarily the case; the degree to which these events are experienced as traumatic may be mediated by age, severity of illness, abruptness of onset, religious faith, and individual interpretation [32]. Among individuals who neither experience an acute emotional response nor interpret a potential stressor as extremely disturbing and frightening, the likelihood of developing PTSD is very low [32-36].

In addition to relying primarily on screening tools, a majority of investigations failed to assess for previous or intervening trauma, although such information is highly relevant in determining both the genesis of PTSD symptoms and the unique contributions of ICU treatment to the development of PTSD. Data suggest that a majority of community-dwelling individuals have been exposed to at least one traumatic event during their lifetime [37] and that those individuals with chronic diseases such as HIV, diabetes, and musculoskeletal disorders (conditions common among ICU cohorts) have unusually high levels of trauma exposure [38-40]. Whether the PTSD symptoms endorsed in the studies to date are primarily a function of ICU-related events or instead are influenced by other traumatic exposures is a crucial question, but one that (in part due to the limitations of current research) cannot be answered.

Yet another limitation of research on PTSD and critical illness pertains to sampling issues. In studies of PTSD in more established populations (that is, combat survivors, victims of sexual assault, and patients with cancer), sample sizes are often quite large and patients are in many cases relatively homogenous. In contrast, the largest study of PTSD following critical illness contained fewer than 150 patients at follow-up, and the majority of investigations consisted of fewer than 50 patients at follow-up and included patients with substantial differences with regard to key characteristics, including the time to PTSD assessment. These issues, along with consistently and strikingly low follow-up rates, raise questions about the generalizability of study findings and the degree to which study participants are representative of typical critically ill populations. It may be, for example, that high-functioning ICU survivors without psychological sequela might conclude that the study participation is of little value to them and thus decline, or that subjects with PTSD might be particularly inclined to participate as a way of seeking help. Alternatively, it may be that some ICU survivors with PTSD may be less likely than their ICU counterparts to participate because the intense emotional distress they experience precludes them from doing so.

### Critical illness as a traumatic stressor

Although the experience of critical illness is undoubtedly stressful, aspects of this experience differ in nature from more traditionally defined and widely studied 'traumas' such as severe burns, automobile accidents, sexual assaults, and exposures to combat. For example, ICU patients are frequently unaware of the degree of life-threat their illness poses until after the illness is largely resolved. Additionally, the development of critical illness is frequently a continuation or acceleration of a longstanding disease process (for example, patients with chronic obstructive pulmonary disease have an exacerbation of symptoms, necessitating ICU care) as opposed to an abrupt occurrence. Despite these caveats, key factors associated with critical illness may be traumatogenic. These could potentially include the diagnosis of critical illness, the unique stresses often associated with ICU care such as intubation and weaning from mechanical ventilation, and the occurrence of nightmares and delusions. The cumulative effects of these factors could increase the likelihood of developing PTSD, particularly in patients with pre-existing vulnerabilities such as a prior history of trauma exposure or a history of chronic medical illness [41-44].

As others have observed, altered mental status (in the forms of both delirium and coma) is common in the ICU, raising important questions about the role of memory (that is, the ability to remember traumatic events) in mediating the development of PTSD [45]. The importance of specific *explicit memories *(memories pertaining to facts and events, which are accessible to consciousness) [46,47] in the generation and maintenance of PTSD is difficult to overestimate as they are the basis for nightmares, flashbacks, and intrusive thoughts and contribute to symptoms of avoidance and re-experiencing. Current evidence suggests that the absence of episodic memory for a traumatic event is protective against the development of PTSD; a majority of studies have shown that the risk of PTSD is markedly lower in individuals unable to recall a traumatic event than in those with explicit memory for such an event (or events) [48-52]. However, some contemporary theories suggest that PTSDcan develop in patients with impaired consciousness for the following reasons: (a) patients can experience the traumatic event after they regain consciousness, (b) processing occurs at an implicit level during periods of impaired consciousness (that is, due to psychological distress encoded by amygdala activation, re-experiencing of symptoms can occur with any memory of the event), and (c) some people appear to reconstruct memories or experiences from photographs, reports that then 'become memories' that may provide the basis for the generation of PTSD symptoms even in the absence of conscious awareness [34,53-55].

## Conclusion

The relationship between critical illness and PTSD has been assessed in a limited number of studies over the last decade and a half. These studies have varied widely in their aims and methodological rigor but have raised awareness and generated valuable data and important insights. For example, we now recognize that sedation strategies can influence the development of PTSD symptoms. Additionally, more recent evidence suggests that individuals with predominantly factual, as opposed to delusional, recollections of the ICU may be at reduced risk for PTSD. Furthermore, it appears that the presence of premorbid mental health problems increases the likelihood of developing PTSD in survivors of the ICU.

Despite the growing recognition that PTSD may occur following an episode of critical illness, the extent to which it can reliably be considered a threat is unknown, due to the methodological limitations and conflicting results of the current studies. It is highly probable that investigations to date have tended to overestimate PTSD prevalence because of an over-reliance on screening tools (as opposed to diagnostic tools), questionable interpretations of available data, the lack of evaluation of non-ICU-related causes of PTSD, low follow-up rates, and other significant limitations. It is worth noting, in this regard, that the three studies reporting the highest rates of actual PTSD (>50%) had sample sizes of between 11 and 27 patients. Developing conclusions about prevalence on the basis of such limited investigations is both extremely imprudent and inconsistent with sound scientific practice. Nevertheless, PTSD clearly occurs and persists in a subset of ICU survivors.

Continued investigation of PTSD in critically ill populations is vitally important for determining the nature and scope of the problem and evaluating possible interventions. However, the relevance and value of a program of investigation will be limited unless it employs the same methodological rigor that characterizes the study of PTSD in other better-established populations such as combat veterans and cancer patients. To that end, specific guidelines should be adhered to and specific goals aggressively pursued. First, studies focused on PTSD as an outcome should use appropriate diagnostic tools and should focus not only on the identification of symptoms but also on the assessment of clinical significance. Researchers should attempt to use populations sufficiently large and representative so as to determine the approximate prevalence of PTSD in critically ill cohorts. In addition to evaluating prevalence rates, investigators should study rates of symptom remission. Second, the incidence of other potentially relevant historical or intervening traumatic stressors and trait variables (for example, neuroticism and anxiety) should be explored. Third, studies should more fully explore the specific etiologies of ICU-related PTSD, placing particular emphasis on the contributions of factual versus delusional memories to the development of PTSD. Fourth, studies should examine the effects of sedation strategies on the development of PTSD, focusing on the identification of strategies that may be protective against the development of PTSD. Finally, studies should assess specific risk factors for the development of PTSD in ICU survivors, focusing in particular on the identification of modifiable risk factors and potential interventions that might reduce the incidence of PTSD or PTSD symptoms. Understanding the nature of the relationship between critical illness and PTSD is a challenge that demands attention, particularly in an era when mental health professionals are beginning to recognize the significant and sometimes profound costs (interpersonal, vocational, medical, and financial) associated with this psychiatric syndrome.

## Key messages

• PTSD or PTSD symptoms are reported to occur in between 5% and 63% of ICU survivors, and key risk factors include duration of hospital and ICU stays, duration of ventilation, pre-existing psychiatric history, and the presence of delusional memories.

• Reported rates of PTSD prevalence following the ICU tend to be extremely high relative to other trauma populations, including medical and surgical patients, and are likely to be overestimates.

• Studies of PTSD following critical illness are characterized by significant methodological shortcomings, which raise key questions about the actual prevalence rates of PTSD and the generalizability of study findings.

• Future studies on PTSD should be more methodically rigorous and should use larger and more homogeneous samples while also employing comprehensive diagnostic, as opposed to screening, instruments.

## Abbreviations

ARDS = acute respiratory distress syndrome; DSM-IV = *Diagnostic and Statistical Manual of Mental Disorders, Fourth Edition*; DTS = Davidson Trauma Scale; ICU = intensive care unit; IES = Impact of Events Scale; PTSD = post-traumatic stress disorder; PTSS = post-traumatic stress symptoms; PTSS-10 = Post-Traumatic Stress Scale-10 for the Intensive Care Unit; SCID = Structured Clinical Interview for the *Diagnostic and Statistical Manual of Mental Disorders, Fourth Edition*.

## Competing interests

The authors declare that they have no competing interests.

## Authors' contributions

JCJ conceived of the manuscript, performed the literature review, and was primarily responsible for writing the manuscript. RPH assisted in the conception of the project and in the writing and drafting of the manuscript, including the creation of tables. SMG assisted in performing the literature review and in the writing and drafting of the manuscript, including the creation of tables. ROH assisted in performing the literature review and the writing, drafting, and editing of the manuscript, including the creation of tables. TDG assisted in the writing, drafting, and editing of the manuscript. EWE contributed to the conception of the project and assisted in the writing and drafting of the manuscript. All authors read and approved the final manuscript.
